# Case Report: A complex congenital bilateral multidirectional glenohumeral hyperlaxity with instability: surgical, anatomical, and forensic insights

**DOI:** 10.3389/fsurg.2025.1578404

**Published:** 2025-06-02

**Authors:** Marco Capuzzo, Fernando Henrique Mizuno, Gaetano Maci, Luca Carboni, Aron Emmi, Veronica Macchi, Raffaele De Caro, Andrea Porzionato, Rafael Boscolo-Berto

**Affiliations:** ^1^ Orthopedic Unit, Villa Regina Hospital, Bologna, Italy; ^2^ M2Clinic, Treviso, Italy; ^3^ Medicine and Surgery Student, University of Bologna, Bologna, Italy; ^4^ Department of Neurosciences, Institute of Human Anatomy, University of Padova, Padova, Italy

**Keywords:** surgery, multidirectional instability, orthopedics, anatomy, morphology, glenohumeral joint, legal medicine

## Abstract

Multidirectional instability (MDI) of the shoulder joint involves the looseness of the joint capsule in multiple directions, resulting in difficulties in keeping the head of the humerus centered within the glenoid fossa. There is still considerable debate about the optimal treatment approach, ranging from conservative management to surgical intervention and it is even more challenging for complex or bilateral cases. An 18-year-old male with a rare congenital bilateral multidirectional glenohumeral hyperlaxity and instability is reported. After excluding other medical conditions, the patient was diagnosed with benign joint hypermobility syndrome. Despite undergoing four months of conservative treatment with physical therapy, there was no significant improvement, leading to the decision for bilateral surgical intervention. The procedure combined an autograft Posterior Bone Block procedure with the Arthroscopic Subscapularis Augmentation (ASA) Technique to enhance anterior stability. The latter involved a tenodesis of the superior third part of the subscapularis tendon. The fixating hole was drilled at the top position of the glenoid edge, and the insertion on the subscapularis tendon was positioned inferiorly to the superior border of the tendon. After surgery, an accelerated post-operative rehabilitation protocol for each shoulder was implemented. At the one-year follow-up after the second surgery, the patient demonstrated substantial improvements in shoulder stability and functional outcomes. The Constant Shoulder Score (CSS) improved from 53 to 77 for both shoulders, indicating a 45.3% improvement and progression from “Moderate” to “Good” function. Similarly, the American Shoulder and Elbow Surgeons (ASES) Orthopaedic Scores improved from 58.33 to 88.32 for the right shoulder (51.4% improvement) and from 61.65 to 94.99 for the left shoulder (54.1% improvement), reflecting a transition from “Fair” to “Good” and “Excellent” function, respectively. Importantly, no short- or medium-term adverse events were reported, and the patient achieved a full return to normal activities. The combination of autograft Posterior Bone Block and ASA techniques has proven to be a successful option in this case for restoring function and stability, even in rare and complex cases of congenital bilateral multidirectional glenohumeral hyperlaxity and instability. Nonetheless, in these complex cases, critical surgical, anatomical, and forensic issues should be carefully considered.

## Introduction

The majority of glenohumeral dislocations occur in the anterior-inferior-medial direction (95%) and less frequently in the posterior direction (3%). Multidirectional dislocations are rare, accounting for up to 1% (1). Multidirectional instability (MDI) is a shoulder joint condition that causes patients to experience looseness of the joint capsule in multiple directions, resulting in difficulties in keeping the head of the humerus centered within the glenoid fossa (2). MDI can develop in patients with congenital joint laxity who have experienced repetitive minor injuries or one or more major injuries to the shoulder (3). The accurate incidence of MDI remains largely unknown due to the variability of classification systems and the complexity involved in diagnosing the disease (4). The condition is most frequently observed during the second and third decades of life, becoming increasingly rare in individuals over the age of 40. This decline is attributed to the natural decrease in tissue flexibility and the physiological stiffening of the shoulder joint with age. In young athletes, the incidence of MDI can reach approximately 10% (5). Although MDI is more common in sedentary women with underdeveloped musculature, it also affects athletes of both genders (4). De Martino and Rodeo evidenced that for swimmers generally the age, years of training and the level of competition are proportional to the incidence of MDI in these athletes. It's worth noting that asymptomatic joint laxity is present in around 10%–30% of the population, with laxity in a single joint being more common than generalized hypermobility. Joint laxity typically decreases steadily in boys, but peaks around age 15 in girls (6). Inactive individuals, particularly young women with poor muscular development and patients with large rotator cuff tendon tears, are at higher risk of developing MDI due to deficiencies in the muscles that stabilize the humeral head (7). MDI can develop in individuals who are active or participate in sports that require repetitive overhead movements, such as gymnastics or tennis (8, 9). Athletes under the age of 40 are more commonly affected by this condition, as tissues around the shoulder naturally stiffen with age, making it less likely to occur after that age. A proper balance between the scapulothoracic stabilizers and the rotator cuff is necessary for maintaining normal joint function; imbalances in these forces may occur due to traumatic events, such as a tackle during a rugby or soccer match, which can lead to anterior dislocation of the glenohumeral joint (10–12). Frequently, the initial event that triggers shoulder instability is repetitive overhead activity, which may not necessarily cause an identifiable episode of instability but does result in pain. This pain leads to guarding the affected shoulder, which over time, weakens the muscles and affects neuromuscular coordination (13, 14). This cycle continues and leads to a worsening of the condition, where the patient becomes increasingly prone to shoulder instability and experiences more severe pain as the shoulder becomes weaker and more dysfunctional. The younger the patient, the higher the likelihood of recurrent shoulder dislocation following traumatic injuries (15).

Although significant progress has been made in the understanding of MDI, there is still considerable debate about the optimal treatment approach for MDI, with some clinicians advocating for conservative management, while others propose surgical intervention. Nonetheless, exercise-based rehabilitation plays a crucial role in managing multidirectional shoulder instability, particularly in patients without a history of prior injury. However, rehabilitation is not without its challenges. Patients often face difficulties achieving consistent improvements due to the complexity of the condition, which may include altered movement patterns, imbalanced muscle activity, and poor neuromuscular control. Additionally, tailoring the type of exercises, training volume, and intensity to match the patient's current condition requires careful assessment and ongoing adjustments.

When rehabilitation alone fails to provide satisfactory outcomes, often due to limited progress or persistent symptoms, arthroscopic techniques to reduce the volume of the joint capsule may be considered. Even after surgical intervention, the rehabilitation process remains essential. Individuals with multidirectional instability must focus on retraining proper scapular positioning, enhancing proprioception, and improving neuromuscular control of the rotator cuff and periscapular muscles to restore function and prevent recurrence (16). A unique case of complex congenital bilateral multidirectional glenohumeral hyperlaxity and instability that was successfully treated through surgical intervention is here reported, delving into the anatomical, surgical, and forensic insights.

## Case report

An 18-year-old male student, right-handed, has been experiencing constant and lingering pain in the right shoulder, coupled with subluxation, over the past six months. The patient reported that he had pre-existing bilateral shoulder instability during daily activities, characterized by a sensation of joint mobility even during simple movements. The patient initially perceived this unusual mobility as normal and did not consider it a pathological condition until symptoms progressively worsened. Although the dislocations were partial and spontaneously reduced, he reported progressive pain and a sensation of persistent instability. This condition was further aggravated after a traumatic event during a casual soccer match, during which the patient recalled a worsening of symptoms, especially in his dominant right shoulder, ultimately leading him to seek medical evaluation. No rolling over both shoulders during the incident was reported.

### Diagnostic assessment

The diagnostic workup for this case of suspected bilateral multidirectional instability (MDI) involved a comprehensive clinical evaluation followed by targeted imaging studies.

During the clinical interview, the patient reported experiencing increased instability in both shoulders during certain overhead activities, such as reaching and lifting above shoulder height. The patient had no history of prior shoulder surgeries, and cervical spine disease, os acromiale, and thoracic outlet syndrome were ruled out. The absence of motor or sensory deficits, along with a normal cervical spine examination, excluded neurological causes. Moreover, hyperlaxity syndromes were excluded basing on Beighton's criteria, and no signs relating to Ehler-Danlos syndrome, Marfan syndrome, osteogenesis imperfecta were detected. Additionally, the patient reported no familial history of shoulder laxity, instability, or related connective tissue disorders. A Jobe test was performed on both shoulders, revealing positive signs of shoulder instability. While the Jobe test is typically used to evaluate the supraspinatus muscle, its performance in this case was sufficient to indicate the shoulder's instability. The patient did not exhibit the sulcus sign in either shoulder, nor any motor or sensory deficits. Posterior dislocation was observed in the right shoulder, while anterior dislocation was noted in the left shoulder.

### Right shoulder assessment

On the right shoulder, the Contrast-enhanced MRI scan showed a rupture in the middle glenohumeral ligament and a SLAP lesion (Superior Labrum from Anterior to Posterior) and a type III capsular insertion. These results underscore the complex and severe nature of the patient's condition. Furthermore, it was possible to ascertain that the patient exhibited a congenital ligamentous laxity, and that the glenoid labrum appeared hypotrophic. These pre-existing anatomical factors likely contributed to the patient's predisposition to instability and to the worsening of symptoms following trauma, emphasizing the multifactorial origin of the injury and the need for a comprehensive therapeutic approach. The extra-articular section of the tendon of the long head of the biceps brachii muscle was unaffected. A small notch of the humeral head in the dorsal planes, externally, indicated stabilized outcomes of a Hill-Sachs type impact bone injury. On clinical examination, the rotator cuff was confirmed to be intact, consistent with the imaging findings. However, significant capsular and soft tissue laxity of the shoulder was identified, which contributed to the overall instability. The patient also reported subjective apprehension during overhead movements. These findings reinforce that, despite preserved muscle and tendon integrity, the underlying capsular insufficiency and generalized soft tissue laxity were the primary factors driving the symptomatic instability.

### Left shoulder assessment

On the left shoulder, a type II capsular insertion was identified, accompanied by an anomalous extra-capsular diffusion path in the sub-coracoid area and deep to the subscapularis. Notably, a capsular lesion was appreciated at this level, with an absence of recognizable capsule in the antero-superior area. Ruptures of the superior and middle glenohumeral ligaments, with the latter being unlaminated, were detected. The glenoid labrum exhibited inhomogeneity from 12 o'clock to 1 o'clock and also appeared hypotrophic. These findings underscored the extensive compromise of static stabilizers in both shoulders, reinforcing the need for a comprehensive surgical approach to restore stability. Synovial thickenings in the rotator interval were also noted, along with uneven thickness of the anterior band of inferior glenohumeral ligament, suggestive of a fibrotic-cicatricial component. No MRI signs indicated involvement of the periosteal bone component. All the clinical analysis outcomes are summarized in Table 1, and all the imaging findings in clinical analysis are presented in Table 2.

**Table 1:** Clinical analysis outcomes.

Findings	Right shoulder	Left shoulder
Patient-reported Symptoms	Instability during certain movements, particularly troughing activities	Instability during certain movements, particularly troughing activities
History of prior surgeries	No	No
History of cervical spine disease	No	No
History of os acromiale	No	No
History of thoracic outlet syndrome	No	No
Hyperlaxity Syndromes (Beighton's criteria)	Excluded	Excluded
Marfan syndrome	Not detected	Not detected
Ehler-Danlos syndrome	Not detected	Not detected
Osteogenesis imperfecta	Not detected	Not detected
Jobe Test	Positive signs of instability	Positive signs of instability
Sulcus Sign	Absent	Absent
Neurological Deficits	None	None
Dislocation	Posterior	Anterior

The table summarizes the outcomes of the clinical analysis.

**Table 2:** Images findings in clinical analysis.

Imaging findings	Right shoulder	Left shoulder
SGHL	Normal	Ruptured
MGHL	Ruptured	Ruptured (unlaminated)
IGHL	Atrophy of the anterior inferior glenoid labrum and a lesion of the inferior glenohumeral ligament	Uneven thickness in the anterior band (fibrotic-cicatricial component)
SLAP lesion	Present	Not observed
Glenoid labrum	Hypotrophic	Hypotrophic and inhomogeneous labrum (12–1 o'clock)
Periosteal bone involvement	Not observed	Not observed
Fibrotic-cicatricial component	Not observed	Present
Synovial thickening	Not observed	Observed in the rotator interval
Type of scapular insertion of the capsule	Type III	Type II
Capsule lesion	Not observed	Capsular lesion observed and absence of recognizable capsule in antero-superior area
Anomalous extra-capsular diffusion path	Not observed	Sub-coracoid area and deep to subscapularis
Rotator cuff components	Normal	Normal
Hill-Sachs lesion	Observed	Not observed
Long head of biceps brachii tendon	Normal	Normal

SGHL, superior glenohumeral ligament; MGHL, middle glenohumeral ligament; IGHL, inferior glenohumeral ligament; SLAP lesion, superior labrum from anterior to posterior lesion.

### Therapeutic intervention

Initially, a cautious approach involving physical therapy was taken to enhance the dynamic control and positioning of the humeral head in the glenoid. A rehabilitation exercise regime was put in place to concentrate on correcting scapulothoracic dyskinesia and strengthening the dynamic stabilizers of the glenohumeral joint. Unfortunately, pain, laxity, instability persisted, and satisfactory results were not observed.

A thorough and systematic evaluation of the diagnostic methods, clinical findings, and imaging results was conducted prior to proceeding with the definitive therapeutic decision. The clinical assessment incorporated not only patient history and physical examination, but also advanced imaging studies, including contrast-enhanced MRI. After the exclusion of differential diagnoses—such as neurologic disorders, cervical radiculopathies, hyperlaxity syndromes like Ehlers-Danlos and Marfan syndromes—the persistence of instability symptoms despite adherence to a structured and prolonged conservative rehabilitation program indicated a poor prognosis with non-surgical management alone.

**Figure 1:**
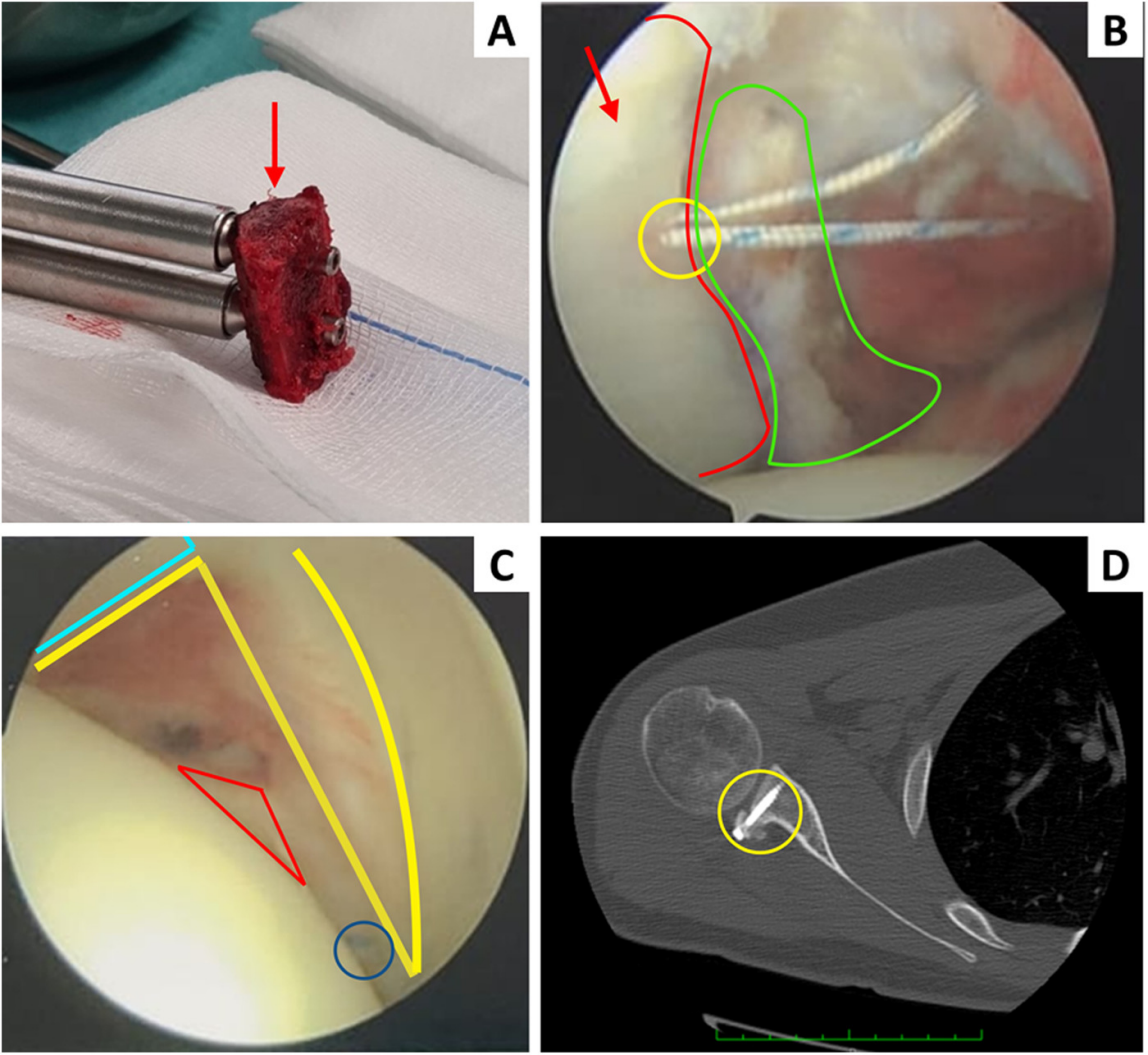
**(A)** Iliac crest autograft is sourced and ready for reimplantation. The red arrow indicates the iliac crest graft, which was modelled and positioned using the Arthrex®guide for Latarjet surgery. **(B)** Bankart lesion in the left shoulder from an arthroscopic perspective. The picture provides a frontal view of the posterior part of the left glenohumeral joint. The surgical lip and the posterior capsule will be reattached using a FiberTak® Soft Anchor (Arthrex®) previously inserted into the bone (yellow). The articular surface of the glenohumeral joint is highlighted in red, while the iliac crest graft is shown in green. **(C)** This is an arthroscopic view of the ASA Technique being performed on the left shoulder. The long head of the biceps brachii is marked in light blue, the anterior part of the glenoid lip is in yellow, the subscapularis muscle following the ASA technique is marked in red, the FiberTak® Soft Anchor (Arthrex®) is circled in dark blue. **(D)** CT scan of the right shoulder following bone block repair and ASA Technique. Following CT evaluation, the graft (yellow circle) has been positioned posteriorly to the glenoid using two cannulated Arthrex®screws.

In view of the documented failure of conservative treatment, the decision to proceed with surgical intervention was carefully deliberated within a patient-specific framework. This approach ensured a targeted and individualized therapeutic strategy, aiming not only to correct the mechanical deficiencies but also to optimize long-term functional outcomes. The overall therapeutic decision-making pathway, including the progression from conservative rehabilitation attempts to surgical treatment, and the postoperative rehabilitation is comprehensively summarized in Supplementary Material Figure A. Consequently, the surgery was indicated and performed on the right shoulder (2020) and left shoulder (2022) with the same operating procedures. To meticulously assess the glenoid-humeral shoulder joint, an arthroscopic examination was conducted. Utilizing a 30° 4.5-mm arthroscope introduced through the rotator interval, the examination took place with the patient positioned in the beach-chair posture under general anesthesia and an interscalene block. Throughout the arthroscopy, it was observed that the subscapularis, supraspinatus, infraspinatus, and teres minor tendons remained intact. The procedural steps involved creating a working posterior portal through the “soft spot” posteriorly, approximately 2 cm inferior and medial to the posterolateral aspect of the acromion, addressing the capsular lesion. Additionally, another working portal was established anteriorly, medial to the viewing portal. Subsequently, a Posterior Bone Block (PBB) surgery was executed, utilizing an autograft sourced from the iliac crest. The iliac crest graft harvesting involved an incision of approximately 5 cm on the lateral part of the iliac crest region. The obtained bone graft was meticulously shaped to conform to the posterior aspect of the glenoid, and fixation was achieved using two metallic screws as shown in Figure 1A, which demonstrates the iliac crest autograft sourced, modelled, positioned using the Arthrex® guide for Latarjet surgery and ready for reimplantation. After the iliac crest graft implantation, the Arthroscopic Subscapularis Augmentation Technique (ASA Technique) was systematically applied to enhance anterior stability, consistently performed on both shoulders. This method involves a tenodesis of the superior third part of the subscapularis tendon. The fixating hole was drilled at the top position of the glenoid edge (at 2 o'clock on the glenoid surface), and the insertion on the subscapularis tendon was positioned 5 mm inferior to the superior border of the tendon as shown in Figure 1B. Then, the tendon was fixated to the head of the scapula using a Knotless 1.8 FiberTak® Soft Anchor (Arthrex®) on each shoulder. The last step is illustrated in Figure 1C, which shows an arthroscopic view of the ASA Technique being performed on the left shoulder. The long head of the biceps brachii is highlighted in light blue, the anterior part of the glenoid lip in yellow, the subscapularis muscle following the ASA technique in red, and the FiberTak® Soft Anchor (Arthrex®) is circled in dark blue.

An accelerated post-operative rehabilitation protocol for each shoulder was implemented for each shoulder. This protocol was designed based on the framework and aligned with the evidence-based recommendations outlined by Goldenberg et al. (17).

The rehabilitation process was structured into five distinct phases: Phase I: Protection phase; Phase II: Active range of motion and muscle endurance; Phase III: Initial resistance strengthening; Phase IV: Advanced muscular strengthening and power; Phase V: Return to sport.

This involved immobilization of the shoulder for 3 weeks (Phase I), followed by simple motion exercises for 4 weeks (Phase II). During this phase, patients were advised to perform daily activities while limiting shoulder abduction and external rotation to 45 degrees. At 8 weeks, gentle stretching was introduced to enhance the range of motion, and after 12 weeks, strengthening exercises were initiated (Phase III and IV) (17).

All the details about the Rehabilitation Protocol used are presented in Table 3.

**Table 3:** Posterior labral repair rehabilitation protocol used.

Phase	Category	Details
Phase I (Protection phase) 0–3 weeks post-Op rehabilitation	Physical therapy	–
	Precautions	Continue to wear sling (all components) daily except during home exercises and physical therapy.
	Sling Use	Must wear sling (all components) at all times, except during home exercises and physical therapy
	Range of motion	Encourage pendulum exercises at home 3x/day
		Full elbow flexion and extension
		Wrist ROM exercises
		Can not extrarotate the shoulder
	Home instructions	Keep surgical dressings clean and dry
		Change surgical bandages on the 2nd day after surgery (keep covered until first clinic visit)
		Can bathe on the 2nd day after surgery (do not scrub, soak, or submerge the incisions)
		Must sleep in the sling
		TECAR Therapy as instrumental therapy
Phase II (active range of motion and muscle endurance) 3–7 weeks post-Op rehabilitation	Physical therapy	3 times per week (40 min per session)
	Precautions	Continue to wear sling (all components) daily except during home exercises and physical therapy.
		Keep arm in front of body when out of the sling/immobilizer. Do not reach the arm behind the back!
	Sling Use	Must always wear sling (all components), except during home exercises and physical therapy
	Range of motion	Passive ROM under ATC or PT supervision (no pulley exercises at home without supervision):
		Flexion to 60°
		Extension to neutral (0°)
		Abduction to 90°
		External rotation to 45°
		Internal rotation to neutral with arm at side (0°)
	Home instructions	Can submerge incision in water after first post-op visit only when incision is completely healed
		No lifting with operative shoulder
		Do not support bodyweight with operative shoulder
		Do not reach the arm behind the back
Phase III (initial resistance strengthening) 7–10 weeks post-Op rehabilitation	Physical therapy	3 times per week (40 min per session)
	Precautions	Keep arm in front of body when out of the sling/immobilizer. Do not reach the arm behind the back!
		Do not perform over-head movements
		Do not perform any repetitive movement with the shoulder
		Do not exceed 2 kg for pull or push movements
	Sling Use	Continue to wear sling (all components) daily except during home exercises and physical therapy. May discontinue sling use at night while sleeping but avoid internal rotation
	Range of motion	Passive ROM with pulleys or other assistive device:
		Flexion to 90°
		Abduction to full as tolerated
		Extension to 30°
		External rotation to 45° with arm abducted to 90°
		External rotation to full as tolerated with arm at side
		Internal rotation to 30° with arm abducted to 90°
		Active Assisted ROM with wand or other assistive device (standing or supine):
		Wall walks in flexion and abduction
		Isometric Exercises
	Home instructions	No longer need to wear the sling
		No lifting, pulling, or pushing greater than 2 pounds
		No overhead work
		No repetitive motions with the shoulder
Phase IV (advanced muscular strengthening and power) 10–13 weeks post-Op rehabilitation	Physical therapy	3 times per week (40 min per session)
	Precautions	Limit internal rotation to 45° until 12 weeks post-op
		Do not perform any repetitive movement with the shoulder
		Do not exceed 3 kg for pull or push movements
	Sling use	Discontinue sling/immobilizer at 6 weeks post-op
	Range of motion	Advance active ROM to full as tolerated (except IR)
		Limit IR to 45° with both arm at side and abducted to 90°
		Regain normal glenohumeral:scapular 2:1 motion
		Isometric Exercises
Phase IV (advanced muscular strengthening and power) 10–13 weeks post-Op rehabilitation (continued)	Range of motion (continued)	Advance shoulder strengthening exercises to include UBE and wall push-ups
		Standing flexion, extension, abduction, and scaption with thumb down with dumbbells or Therabands
		Standing IR and ER with Theraband with arm abducted 25° at side (with pillow or towel)
		Advance scapular strengthening exercises
		Begin neuromuscular control exercises
	Home instructions	No lifting, pulling, or pushing greater than 3 kg
		No overhead work
		No repetitive motions with the shoulder
Phase V (return to sport) after 13 weeks post-Op rehabilitation	Physical therapy	3 times per week (40 min per session)
	Precautions	Limit internal rotation to 45° until 12 weeks post-op
	Sling Use	Continue to wear sling (all components) daily except during home exercises and physical therapy. May discontinue sling use at night while sleeping but avoid internal rotation
	Range of motion	Full active ROM as tolerated (except IR)
		Limit IR to 45° with both arm at side and abducted to 90°
		Continue scapular strengthening and isotonic rotator cuff strengthening exercises until full ROM is restored
		Continue dumbbell exercises
		Continue neuromuscular exercises
		sokinetic strengthening with 60° block
	Home instructions	No lifting, pulling, or pushing greater than 7 pounds
		No overhead work
		No repetitive motions with the shoulder

Follow-up visits were conducted for monitoring, and a comprehensive one-year physiotherapy plan was recommended. After a period of two months from the last surgery, the patient was able to resume normal activities (Phase V). Additionally, CT scan results showed no bone lesions related to the procedures. Correct positioning of the iliac crest autograph (yellow circle) can be observed in the CT scan of the right shoulder following bone block repair and ASA Technique in Figure 1D. Another month later, the patient had fully recovered from the surgery for both shoulders.

During a one-year follow-up after the last surgery, it was observed that the patient's shoulders stability had improved significantly, as shown by the Constant Shoulder Score and The American Shoulder and Elbow Surgeons Orthopaedic Scores (Table 4 and Supplementary Material Table B). The patient was also more confident in performing movements that were previously difficult.

**Table 4:** The constant shoulder score and the American shoulder and elbow surgeons (ASES) orthopaedic scores (53).

Clinical score	Right shoulder (1st surgery) score results		Left shoulder (2nd surgery) score results	
	Before surgery	After surgery	Before surgery	After surgery
Constant shoulder score	53	77	53	77
ASES orthopaedic scores	58.33	88.32	61.65	94.99

CSS (constant shoulder score): poor (0–30 points), moderate (31–60 points), good (61–80 points), and excellent (81–100 points). ASES orthopaedic scores: poor (0–50 points), fair (51–75 points), good (76–90 points), and excellent (91–100 points).

Supplementary Material Table B presents a detailed breakdown of the Constant Shoulder Score (CSS) subdomains assessed before and after each sequential surgical intervention, highlighting the progressive functional improvements achieved through the surgical procedures.

Before the surgeries, the patient exhibited moderate pain in both shoulders, limited recreational/sports activities, and reduced abduction strength (1–3 lbs), with arm elevation restricted up to neck level. Although the range of forward flexion remained relatively preserved (121°–150°) in both shoulders, the lateral elevation presented a slight decreased in range (from 151°–180° to 121°−150°), the qualitative descriptors of external and internal rotation suggested functional limitations, with external rotation characterized by the elbow positioned forward when reaching the head. The overall Constant Shoulder Score at baseline (prior to surgery) was 53 for both shoulders.

Postoperatively, the patient reported complete resolution of pain, full return to recreational/sports participation, demonstrating a substantial recovery of shoulder function. Importantly, abduction strength improved to 7–9 lbs, and arm positioning advanced from “up to neck” to “above head” levels, indicating restored overhead capabilities. Internal rotation remained stable, reaching the interscapular T7 level.

The Constant Shoulder Score increased markedly to 77 for both shoulders, moving from “moderate” to “good” functional categories postoperatively. Supplementary Material Table B not only corroborates the patient's subjective clinical improvements but also objectively validates the functional gains across key domains of the CSS framework, aligning with the overall success reported at the one-year follow-up.

## Discussion

Patients with MDI experience looseness of the shoulder joint capsule in multiple directions, causing difficulty in keeping the humerus centered in the glenoid fossa. It's important to note that glenohumeral laxity and instability are not interchangeable terms (2). Instability refers to dysfunction, which can be voluntary or involuntary, unilateral or multidirectional, and caused by trauma or non-trauma. While patients with ligamentous laxity may not experience any symptoms and require no treatment, those with instability are symptomatic by definition and require medical intervention. However, due to the several definitions of MDI in medical literature, it can be challenging to classify patients with this condition. To better clarify these aspects, a streamlined recall of the anatomical region of the shoulder joint with its complex and delicate anatomical-functional balance is useful. The shoulder joint, also known as the glenohumeral joint, is not tightly bound by a thick capsule, but rather by surrounding muscles and ligaments. The shoulder's high degree of mobility is largely due to the glenoid's shallow depth and the limited contact between the glenoid and the humeral head. Only a quarter of the humeral head's surface touches the glenoid. The labrum, a fibrocartilaginous ring attached to the outer rim of the glenoid, provides some additional depth and stability to the joint. The glenohumeral joint is particularly prone to injury and instability due to its shallow and small surface area. To ensure stability, surrounding muscles and ligaments must provide extrinsic support. The glenohumeral ligaments, which consist of the superior, middle, and inferior glenohumeral ligaments, serve as the main static stabilizers. Meanwhile, the rotator cuff, composed of four muscles (supraspinatus, infraspinatus, subscapularis, and teres minor), serves as the primary dynamic stabilizer. This cuff of muscles encircles the head of the humerus, securing it within the glenoid while allowing for a full range of motion. The supraspinatus muscle has its origin in the supraspinous fossa situated on the superior-posterior part of the scapula, which is located above the scapular spine. It then attaches to the greater tubercle of the superior-lateral humeral head, positioned just behind the biceps tendon. Prior to its insertion, the muscle, along with the subacromial bursa, traverses a narrow space between the acromion process and the humeral head. If the shoulder is abducted, the supraspinatus muscle may become impinged between the greater tubercle and the acromion bone. This can result in injury and compression of the muscle and tendon, particularly during activities that involve frequent arm-raising with this kind of squeezing action. The subacromial bursa is essential in shielding the rotator cuff tendons from harm caused by pressure and friction from the acromion bone. Apart from the supraspinatus, three other muscles contribute to the rotator cuff. The infraspinatus muscle arises from the infraspinatus fossa located at the back of the scapula and inserts on the lateral part of the humeral head at the greater tuberosity, just behind the supraspinatus. The teres minor muscle arises from the inferior-posterior scapula and blends with the infraspinatus muscle to attach to the greater tuberosity. The subscapularis muscle, the largest of the four cuff muscles, arises from the anterior scapula, below the coracoid, and attaches to the anterior humeral head at the lesser tuberosity. The rotator cuff holds the humeral head in the glenoid fossa, also acting as a counterbalance to the elevating forces of the deltoid muscle and other muscles that impact the humerus.

When a patient's shoulder can move slightly out of its socket in one or more directions, without causing any pain or discomfort, this is known as laxity without instability. No treatment is necessary in such cases. If the patient experiences pain, weakness, fatigue, or numbness in the shoulder or upper arm, this might suggest laxity with instability. Those with MDI of the glenohumeral joint may exhibit laxity in both shoulders, but only the symptomatic shoulder is considered unstable (18, 19).

In this case, a complex and rare condition was observed: double congenital Bilateral Multidirectional Glenohumeral Hyperlaxity with Instability. The patient did not report any overhead sports-related microtraumas, and specific pathological forms such as Ehler-Danlos syndrome, Marfan syndrome, and osteogenesis imperfecta were ruled out (20–22). Additionally, hyperlaxity syndromes using Beighton's criteria were also excluded (23). After a thorough examination of all these factors, the diagnosis of an underlying benign joint hypermobility syndrome was made (20).

The optimal approach to addressing MDI in patients begins with a thorough assessment of their unique requirements. Notably, in some cases, a degree of flexibility may prove advantageous, such as for swimmers who depend on shoulder mobility for a competitive edge (24).

As such, there is no one-size-fits-all formula for treating MDI. Rather, the treatment approach is tailored to the patient's individual needs and expectations (24). It is recommended to approach the treatment of MDI with a gradual and cautious approach, beginning with conservative methods and to monitor the treatment efficacy using instrumented techniques such as the Constant Shoulder Score (CSS) and the American Shoulder and Elbow Surgeons (ASES) Orthopaedic Scores (4, 16, 25), as shown in Supplementary Material Figure A, which presents a timeline of the patient's diagnostic process, treatment milestones, surgical interventions, and rehabilitation phases. In the event that these methods do not provide sufficient benefits, surgery may be necessary (16). MDI often occurs following an imbalance between the dynamic and static stabilizers, making it important for the initial treatment to involve physical therapy that targets the strengthening of the dynamic stabilizers in the shoulder (22, 26). This included a plan to address shoulder instability by strengthening the rotator cuff and periscapular muscles through closed kinetic chain exercises. These exercises encourage coordinated movements, leading to greater dynamic stability of the shoulder. To ensure effectiveness, the therapy regimen should be comprehensive and sustained for at least six months before exploring more aggressive options. This extensive approach has been shown to be successful, thanks to the shoulder joint's natural stiffening with age. As skeletal maturity approaches, symptoms of MDI tend to decrease, reinforcing the importance of a prolonged therapy regimen (22, 26, 27).

Despite undergoing physical therapy for a year, there were no significant clinical improvements in this case. Given the persistent bilateral instability, alternative therapeutic strategies were considered. These included prolonged physiotherapy, isolated capsular plication, bone block procedures, or combined approaches. After discussing the long-term prognosis and weighing the potential benefits and risks of residual therapeutic options, the patient decided to proceed with the proposed surgical treatment and provided informed consent. However, considering the documented capsular insufficiency, congenital ligamentous hyperlaxity, and the multifactorial nature of instability involving both the anterior and posterior structures, it was concluded that isolated soft tissue repair alone would likely be insufficient to restore full stability. Therefore, the decision was made to implement a combined surgical strategy.

A Posterior Bone Block (PBB) procedure was chosen to address posterior instability and increase glenoid containment, while the Arthroscopic Subscapularis Augmentation (ASA) Technique was selected to enhance anterior stability by reinforcing the dynamic restraint provided by the subscapularis tendon. This comprehensive dual intervention was aimed at restoring global stability and minimizing the risk of recurrent dislocations, based on the multifactorial nature of the patient's instability. This decision was further supported by the growing body of evidence suggesting that both the ASA Technique and the Posterior Bone Block procedure can be utilized not only as therapeutic interventions for traumatic shoulder instability but also as preventive measures in patients presenting with multidirectional instability and hyperlaxity, especially when conservative treatments have failed (28–30). Considering these findings, the surgical plan also aimed at a preventive stabilization of the contralateral shoulder, which, although initially less symptomatic, exhibited instability features and lacked satisfactory response to non-operative treatments.

### Detailing the surgical approach

In selecting the graft material for the posterior bone block, although alternative graft options such as cadaveric distal tibial allografts could have been considered due to their ease of shaping and potential cartilage surface congruence with the glenoid (31, 32), an autologous iliac crest graft was ultimately preferred in this case. Current literature suggests that while distal tibial allografts offer favorable geometric matching and eliminate donor site morbidity, they also present notable drawbacks, including slower biological incorporation, increased risk of partial resorption, higher costs, and limited long-term clinical outcome data compared to autografts (31–33). In contrast, autologous bone grafts provide superior osteogenic potential, lower immunogenic risk, and well-established durability, with reported low rates of recurrent instability when iliac crest autografts are used for posterior bone block procedures (34, 35). Considering the patient's young age, bilateral involvement, congenital ligamentous hyperlaxity, and the necessity for a structurally robust and reliably integrating graft to support long-term shoulder stability, the iliac crest autograft was deemed the most appropriate and durable choice.

A detailed comparative analysis of different graft options — including iliac crest autograft, scapular spine autograft, distal clavicle autograft, coracoid process autograft, and distal tibial allograft — was performed and is summarized in Supplementary Material Table A. Alternative autologous grafts such as the scapular spine, distal clavicle, and coracoid process were carefully evaluated but ultimately considered suboptimal due to their smaller available bone volume, reduced mechanical strength, and greater potential for compromising adjacent anatomical structures, particularly the acromioclavicular joint when harvesting the distal clavicle (5, 31, 36). Additionally, the use of the coracoid process was excluded not only because of its limited graft volume but also due to the experimental nature of this technique in the context of posterior glenoid reconstruction, with very few cases reported in the literature and lacking robust clinical evidence to support its routine application (5). Therefore, to maximize structural stability, biological integration, and long-term clinical outcomes, the iliac crest autograft was selected as the optimal graft material for this complex and demanding case.

Anterior shoulder instability is a prevalent condition among patients and is often accompanied by antero-inferior hyperlaxity of the capsule. This hyperlaxity can cause elongation of the subscapularis tendon, specifically in its superior third portion. The ASA Technique is a treatment strategically applied to address capsular insufficiency. It works by restoring tension in the coracohumeral ligament, mitigating intrinsic laxity, and fortifying stability. This approach helps to reduce the risk of recurrent dislocations, making it an effective treatment for patients with anterior shoulder instability. Moreover, what the ASA Technique avoids compared to the more widespread Latarjet technique is the appearance of early arthrosis of the operated shoulder, which represents its most obvious clinical advantage.

For this setting where precise tension control is crucial during a low-profile repair, the Knotless 1.8 FiberTak® Soft Anchor (manufactured by Arthrex®) was chosen. This anchor system is designed to provide a strong and secure fixation while minimizing the amount of soft tissue disruption. Its knotless design eliminates the need for knot tying, which reduces the potential for knot-related complications and helps to streamline the overall procedure. With its small size and low profile, the system resulted appropriate in tight anatomical spaces, helping to minimize postoperative discomfort for the patient.

At the one-year follow-up, the results in Table 1 and Supplementary Material Table B demonstrate significant improvements in both the Constant Shoulder Score (CSS) and the American Shoulder and Elbow Surgeons Orthopaedic Scores (ASES) following surgical intervention, underscoring the success of the procedures. For the CSS, both shoulders improved from a pre-surgical score of 53 to a post-surgical score of 77, reflecting a 45.28% increase. Similarly, the ASES scores showed notable gains, with the right shoulder improving from 58.33 to 88.32 (51.41% increase) and the left shoulder from 61.65 to 94.99 (54.08% increase). Both shoulders transitioned to higher functional categories, with CSS improving from “Moderate” to “Good” while approaching “Excellent,” and ASES advancing from “Fair” to “Good” with the right shoulder and from “Fair” to “Excellent” with the left shoulder. These results highlight the effectiveness of the surgical interventions in restoring shoulder function and reducing pain. The slightly greater improvement observed in the left shoulder's ASES score may reflect individual variability in healing or rehabilitation response. Overall, the data underscore the clinical success of the procedures in addressing multidirectional instability and improving patient outcomes. When planning a medical intervention and selecting equipment, it is essential to consider several critical aspects to ensure patient safety. One of the most crucial factors is the risk of medical malpractice allegations that could arise if the intervention is not carried out appropriately.

There are primarily two main adverse events associated with the ASA Technique: capsulitis and joint stiffness. Capsulitis is common in shoulder arthroscopies, especially in females, and is linked to the patient's local inflammatory response to cartilage damage or arthroscopic manipulation (37, 38). Joint stiffness typically manifests as a limitation to external rotation of the shoulder with the arm adducted. Unlike capsulitis, however, the rigidity is often caused by a technical error during surgery when the stitch in the subscapularis is not passed with the arm extra rotated and medially to the glenoid, as it should be. However, no neurological disorders are typically reported following the operation, except for dysfunctional outcomes due to inadequate stretching of the upper limb if the patient is positioned incorrectly on the operating table. These risks can be mitigated by properly positioning the patient on the operating table and ensuring that the equipment selection is based on the patient's specific needs and the type of intervention being performed. This includes ensuring that the equipment is appropriate for the patient's size, weight, anatomical defect, and local condition. Moreover, it is crucial to consider the qualifications and training of the personnel tasked with performing the operation, particularly the orthopaedist. He must be able to operate the chosen devices safely and accurately, meeting the needs that may emerge during the procedure. It implies completion of the surgical learning curve, which can be gained even by practising on cadavers in a safe and controlled environment within reference centres recognized and regulated by strict provisions (39). Recent regulatory developments have highlighted the importance of such cadaver labs for ensuring safe surgical practices (40, 41). These aspects are critical points that must be taken into consideration, as they could lead to medico-legal litigation for alleged medical malpractice, e.g., concerning the surgical indication, planning of the intervention, or technical execution of the procedure.

### Patient perspective

At the time of the first symptoms, the patient, a 17-year-old right-handed male, described a long-standing bilateral shoulder hypermobility, which he initially perceived as normal during daily activities and sports. He did not consider it pathological until the gradual onset of persistent discomfort, subluxations, and increased instability, especially in the right shoulder after a trauma during a soccer match. Although initially tolerable, the pain became more frequent and persistent, rated around 6/10, interfering with daily life and prompting medical evaluation and treatment. In the post-operatory phase, the patient fully adhered to postoperative recommendations, including temporary suspension of sports activities, anticoagulation prophylaxis, structured physiotherapy, and progressive return to gym exercises. He reported a substantial improvement in stability, disappearance of apprehension, and greater confidence during overhead and daily movements. At the time of the last medical visit he feels safer and stronger, particularly during gym training. No new episodes of instability were experienced after the surgeries. Overall, the patient expressed a high level of satisfaction with the surgical outcome, highlighting a significant improvement in his functional abilities, physical well-being, and quality of life.

## Strengths and limitations

This case report presents several strengths, including a detailed clinical characterization, a tailored therapeutic strategy, and a thorough description of surgical procedures based on established anatomical and biomechanical principles. Additionally, the patient's outcome was assessed using validated scoring systems (Constant Shoulder Score and ASES Orthopaedic Scores), reinforcing the reliability of the clinical improvement observed. Nevertheless, some limitations must be acknowledged. First, being a single case report, the generalizability of the findings is inherently limited, and conclusions should be interpreted cautiously when applied to broader patient populations. Second, although the one-year post-surgery follow-up demonstrated significant functional improvements and absence of instability episodes, longer-term monitoring would be necessary to assess the durability of the surgical outcomes and the potential risk of late complications such as recurrent instability or degenerative changes. Finally, the lack of a control group or comparative analysis with alternative surgical techniques prevents definitive conclusions regarding the superiority of the combined Posterior Bone Block and ASA Technique approach. Despite these limitations, this case contributes valuable insights into the management of complex congenital bilateral multidirectional shoulder instability.

### Medicolegal implications

The appropriate diagnosis of Multidirectional Glenohumeral Hyperlaxity with Instability is a critical point to support adequate therapeutic measures, both conservative and operative, according to a progression of the invasiveness and the consequent general repercussions, both in terms of possible results and sequelae, and of possible complications (42). Indeed, legal medicine has experienced a surge in sub-disciplines, resulting in increased scientific contributions to the field in developed nations (43, 44), and one of the growing concerns is medical malpractice with the assessment of clinical cases impacted by alleged medical errors (45). Cases of medical responsibility and liability have become commonplace in every modern physician's practice, leading to economic pressures to insure against risks and a rise in defensive medicine, hence requiring devoted guidelines and suggestions for the professional activity of clinicians (42).

Moreover, cases have been reported in which subjects simulate an illness. Indeed, factitious disorders refer to conditions in which a person intentionally creates or exaggerates illness based on conscious or subconscious symptomatology, such as patients intentionally dislocating their joints, whether it be for attention, or a desire for sympathy (46). The prevalence of factitious disorders is estimated to be up to 5% of all patient encounters, resulting in significant economic and social effects (46). Factitious disorders typically include psychiatric conditions such as somatization, body dysmorphic and conversion disorders, hypochondriasis, Munchausen and SHAFT syndrome (46, 47). In some cases, people may intentionally exaggerate their symptoms for personal gain, such as during assessments for compensation after an injury. This is the case of malingering. The act of malingering involves intentionally feigning or amplifying symptoms or injuries for personal gain, such as securing financial compensation or avoiding work or military service (48–50). Some may even willingly subject themselves to multiple surgeries in order to obtain their benefits, and a small minority of individuals take this deception to an extreme level by undergoing reconstructive surgery despite evidence revealing that their use of the affected limb does not warrant intervention (51). Once the desired outcome is obtained, the affected individual typically ceases the behaviour (46). The correlation between upper extremity ailments and mental health issues could be more significant than what hand surgeons typically acknowledge, and therefore, it is necessary to familiarize ourselves with the indications and manifestations of mental health problems that may manifest in the upper extremity (47, 52). It is not necessary to establish a psychiatric clinic within an orthopaedic clinic. However, it is imperative to demonstrate sensitivity in identifying inconsistencies between clinical findings and objective data, which may be attributable to underlying diseases or ulterior motives. Failure to do so may lead to an inaccurate diagnosis, which can result in suboptimal management of the patient's condition. It's crucial to note that patients who intentionally dislocate their joints tend to have poor outcomes following surgical intervention, and that whether a patient deceives the surgeon, this does not protect the physician from a potential lawsuit (46, 48). Therefore, it is essential to maintain a high level of vigilance, particularly in circumstances where the patient's presentation is atypical or unexpected (50).

## Conclusion

The management of multidirectional instability (MDI) should be personalized, starting with conservative approaches and progressing to surgical intervention if necessary. At the one-year follow-up, significant improvements in both the Constant Shoulder Score (CSS) and American Shoulder and Elbow Surgeons Orthopaedic Scores (ASES) demonstrated the clinical success of the combined surgical techniques, with a 45.28% increase in CSS and over 50% improvement in ASES scores for both shoulders. These results highlight the effectiveness of the autograft Posterior Bone Block and Arthroscopic Subscapularis Augmentation techniques in restoring function, reducing pain, and improving stability, even in complex cases such as congenital bilateral multidirectional glenohumeral hyperlaxity and instability. Nevertheless, it is important to carefully consider critical surgical, anatomical, and forensic issues.

## Data Availability

The raw data supporting the conclusions of this article will be made available by the authors, without undue reservation.

## Supplementary material

Supplementary Image S1 Timeline illustrating the key stages of the patient’s clinical course. The sequence begins with pre-existing bilateral shoulder hyperlaxity and instability, followed by a trauma during a soccer match. A detailed clinical evaluation and diagnostic assessment were performed, followed by an initial attempt at conservative management. In the absence of significant clinical improvement, and despite adherence to rehabilitation protocols, persistent symptoms led to a clinical reassessment and the indication for surgical intervention. Surgical procedures were subsequently carried out on the right shoulder in 2020 and the left shoulder in 2022. Post-operative rehabilitation was structured and progressive, culminating in marked functional recovery, as reflected by substantial improvements in the Constant Shoulder Score (CSS) and the American Shoulder and Elbow Surgeons (ASES) scores during follow-up.
