# Supplementary material for: Case Report: A complex congenital bilateral multidirectional glenohumeral hyperlaxity with instability: surgical, anatomical, and forensic insights
Source: Front Surg. 2025 Jun 2;12:1578404. doi: 10.3389/fsurg.2025.1578404 (PMC12171428; doi:10.3389/fsurg.2025.1578404)
Supplement: Supplementary file 2 [file Table1.docx]

**Supplementary Material - Table A. Comparison of Bone Graft Options for Posterior Glenoid Reconstruction in Shoulder Instability.**

| **Graft** | **Advantages** | **Disadvantages** |
| --- | --- | --- |
| **Iliac Crest Autograft (47,50)** | High structural graft resistance  High bone incorporation rates; low incidence of nonunion or significant graft resorption  Widely studied, consistently improves clinical outcomes with low recurrence (~10%) | Donor site morbidity (hip pain, discomfort) observed in a subset of patients  Requires a second surgical site (hip harvest), increasing surgical time and complication risks  High incidence of glenohumeral osteoarthritis in long-term follow-up (≥18 years of follow-up)* |
| **Scapular Spine Autograft**  **(46,47)** | Harvested through the same posterior shoulder approach, avoiding a second surgical field  Avoids morbidity from distant donor sites like the iliac crest  Biomechanically restores shoulder stability comparably to distal tibial allografts | Limited graft volume and lower structural robustness compared to iliac crest  Risk of suprascapular or axillary nerve injury during posterior scapular dissection  Clinical experience limited to small series; lacks long-term large studies |
| **Distal Clavicle Autograft (51)** | Able to be used with arthroscopic technique and reduces neurovascular and hardware complications of screws  Distal clavicle graft can be easily harvested and causes minimal donor site morbidity  Compresses the labrum and keeps the graft extra-articular  Cost-effective (no bone bank dependency)  Anatomical shaping possible (“congruent arc”) to fit glenoid curvature | Limited bone quantity; resection >15 mm may destabilize acromioclavicular (AC) joint  Sacrifice of AC joint can cause pain or residual instability  No native hyaline cartilage on the graft surface; fibrocartilage coverage develops later |
| **Coracoid Process Autograft**  **(52)** | Autologous graft of reasonable size from the same shoulder (adapted from Latarjet technique)  Case report showed a successful bilateral posterior stabilization via arthroscopic coracoid transfer without neurovascular injury | *There is not an established technique*: only experimental reports available  Technically demanding, neurovascular risks, unfamiliar posterior positioning  Unknown long-term outcomes and safety; use limited to experimental settings |
| **Distal Tibial Allograft**  **(46,47)** | Eliminates donor site morbidity  Anatomically matches glenoid curvature, restores native articular surface  Studies show equivalent biomechanical stability to autograft reconstructions | High cost and dependence on bone bank availability.  Potential slower biological incorporation compared to autografts.  Limited long-term clinical outcome data for posterior shoulder instability |

* The high incidence of glenohumeral osteoarthritis was reported in studies focused on anterior bone block procedures, not posterior reconstructions.
