# Supplementary material for: Case Report: A complex congenital bilateral multidirectional glenohumeral hyperlaxity with instability: surgical, anatomical, and forensic insights
Source: Front Surg. 2025 Jun 2;12:1578404. doi: 10.3389/fsurg.2025.1578404 (PMC12171428; doi:10.3389/fsurg.2025.1578404)
Supplement: Supplementary file 3 [file Table2.docx]

**Supplementary Material – Table B. Comprehensive Evaluation of Constant Shoulder Score (CSS) Subdomains Before and After Sequential Surgical Interventions (53)**

|  | **Before 1^st^ Surgery (Right Shoulder)** | **After 1^st^ Surgery (Right Shoulder)** | **Before 2^nd^ Surgery (Left Shoulder)** | **After 2^nd^ Surgery (Left Shoulder)** |
| --- | --- | --- | --- | --- |
| **Pain** | Moderate | None | Moderate | None |
| **Activity Level - Unaffected Sleep** | Yes | Yes | Yes | Yes |
| **Activity Level - Full Recreation/Sport** | No | Yes | No | Yes |
| **Activity Level - Full Work** | Yes | Yes | Yes | Yes |
| **Arm Positioning** | Up to Neck | Above Head | Up to Neck | Above Head |
| **Strength of Abduction (lbs)** | 1-3 | 7-9 | 1-3 | 7-9 |
| **Forward Flexion** | 121°-150° | 121°-150° | 121°-150° | 121°-150° |
| **Lateral Elevation** | 151°-180° | 121°-150° | 151°-180° | 121°-150° |
| **External Rotation** | Hand to top of Head, Elbow forward | Hand to top of Head, Elbow back | Hand to top of Head, Elbow forward | Hand to top of Head, Elbow back |
| **Internal Rotation** | Interscapular (T7) | Interscapular (T7) | Interscapular (T7) | Interscapular (T7) |
| **Constant Shoulder Score** | 53 | 77 | 53 | 77 |
